# Curiosity in zebrafish (*Danio rerio*)? Behavioral responses to 30 novel objects

**DOI:** 10.3389/fvets.2022.1062420

**Published:** 2023-02-21

**Authors:** Becca Franks, Leigh P. Gaffney, Courtney Graham, Daniel M. Weary

**Affiliations:** ^1^Faculty of Land and Food Systems, The University of British Columbia, Animal Welfare Program, Vancouver, BC, Canada; ^2^Department of Environmental Studies, New York University, New York City, NY, United States; ^3^Fisheries Ecology and Marine Conservation Lab, Department of Biology, The University of Victoria, Victoria, BC, Canada; ^4^Department of Population Medicine, Ontario Veterinary College, The University of Guelph, Guelph, ON, Canada

**Keywords:** exploratory motivation, cognitive enrichment, naturalistic housing, fish welfare, positive welfare, animal agency

## Abstract

Curiosity—the motivation to seek out information—has been studied widely across the animal kingdom. To investigate curiosity in zebrafish we presented 30 novel objects to groups of zebrafish housed in semi-naturalistic tanks (6 tanks; 10 fish/tank; 10-min presentations). During the first 100 s and final 100 s of each object's 10-min presentation period, we recorded each group's: (i) latency to approach the object, (ii) attraction to the object, (iii) social dynamics: agonistic behavior and group cohesion and coordination, and (iv) diving behavior, a stress response in zebrafish. Comparing these behaviors to a 100 s baseline period when no object was present, we tested for neophobia (avoidance of novelty), neophilia (overall attraction to novelty), sustained interest (prolonged attraction to at least some presentations), discriminant interest (certain objects eliciting more attention than others), habituation (loss of interest over time), and alterations to social and stress behaviors. Zebrafish groups readily approached all objects (1 s median latency), were neophilic throughout all object presentations, and showed systematic sustained interest only for some object presentations at the beginning of the study (object presentations 1–10). Over the course of the study, zebrafish also showed signs of habituation such that by the final ten object presentations (21-30), there were no signs of overall sustained interest. During the beginning of the study (object presentations 1–10), we also found evidence for specific object-driven interest, with object ID accounting for 11% of the variability in interest scores (*p* < 0.01), and object-driven interest corresponding to alterations in social behavior: decreased aggression (*p* < 0.02), increased group cohesion (*p* < 0.02), and increased group coordination (*p* < 0.05). By explicitly investigating curiosity in fish, this work reveals that under certain conditions, zebrafish voluntarily engage in cognitive stimulation opportunities. More work is needed to clarify what types of information zebrafish find most rewarding and how long-term exposure to such opportunities may affect fish welfare.

## Introduction

“*Curiosity has its own reason for existing*.” -Albert Einstein

Old Man's Advice to Youth: “Never Lose a Holy Curiosity”. Life Magazine (2 May 1955) p. 64

In its purest form, curiosity refers to the drive to gain information in the absence of clear instrumental goals such as food or shelter (1). From a behavioral biology perspective, curiosity is understood to have evolutionary value in that gaining information may ultimately enable an individual to better exploit resources and manage threats (2). In the immediate-term, however, information-seeking itself can become its own reward and is sometimes prioritized over clear, material gains (1). As such, curiosity has fascinated scholars throughout history, especially in the middle of the 20th century when psychologists and ethologists intensively studied curiosity in species across the animal kingdom (3–5). The past decade has seen a resurgence of interest in the study of curiosity across multiple disciplines, with investigations of curiosity in a range of species including raptors (6), tortoises (7), orangutans (8), honeybees (9), and in one multispecies study, sheep, penguins, lemurs, cockatoos, tortoises, and kangaroos (10).

To understand what features may drive curiosity, Loewenstein (11) proposed that information seeking behavior is elicited when there is an information gap between what is known and what is unknown, without going beyond the cognitive capacity of the individual. Two ways for perceptual objects to have enhanced information potential are by (i) being complex or (ii) being unusual. From a visual perspective, complex objects are those with many parts, shapes, patterns, and/or colors. Previous work has confirmed that visual complexity attracts greater attention from, for example, raptors (6), rats (12), and humans (13). Unusual objects are either ones that are very different from those that the individual has experienced in its own lifetime or, from an evolutionary perspective, those which would not normally be encountered in the ecological niche of that species—in other words, objects that are ecologically implausible. Recent work in orangutans, for example, has shown that they are particularly interested in unusual, ecologically implausible objects (8). Interestingly, some prey animals are also known to be highly motivated to gather information about predators, readily inspecting potential predators despite the evident risks (14, 15). Thus, previous theoretical and empirical work suggests that complexity, unusualness, and predatory potential are all relevant dimensions in eliciting perceptual curiosity.

As these proposed curiosity-eliciting dimensions indicate, experiencing curiosity and curiosity-producing situations are ambivalently charged—they have the possibility of taking on a positive or negative valence, thus complicating the relationship between curiosity and welfare. Human studies have found that heightened curiosity can be associated with positive affect and wellbeing [e.g., (16)], but also frustration (17, 18), sensation-seeking, boredom-avoidance, and substance abuse (19). When measured as exploratory behavior, curiosity's bivalent nature is evident in nonhuman animals as well. On the one hand, opportunities for exploration and exploratory behavior are associated with improved welfare and cognition (20–23). For example, rats maintained in complex social housing were found to have enhanced exploratory tendencies (giving up known rewards and risking aversive conditions in order to explore new spaces) compared to isolated rats in barren cages (24). Conversely, other forms of exploration have been associated with poor animal welfare. For example, undifferentiated exploratory behavior of novel stimuli (i.e., neophilia) in mink was more apparent in individuals housed in non-enriched cages compared to those housed in enriched cages (25, 26).

These and other studies suggests that exploratory behavior in the form of indiscriminate neophilia (quick, fleeting exploratory behavior) may be reflective of an avoidance motivation (escaping aversive or fear-inducing conditions), especially in situations likely to induce boredom. Boredom, a negative state caused by a lack of behavioral opportunities, is identified as a serious welfare concern (27) and an important area of study in animal behavior (28). In contrast, exploratory behavior in the form of free-choice, targeted, and sustained information seeking has been associated with positive wellbeing in both humans and other animals (16, 22, 23). Distinguishing between these two types of exploratory behavior—indiscriminate neophilia vs. targeted and sustained information-seeking—is necessary when attempting to understand the relationship between curiosity and current welfare state.

By investigating curiosity in zebrafish (*Danio rerio*), the present study aimed to provide crucial data for interdisciplinary, comparative work looking at curiosity and welfare across the animal kingdom. Knowledge about curiosity in zebrafish represents a particularly useful starting place for studying curiosity in fish because it can indicate whether similar issues may be at stake for other species of fish, including the welfare of fish in aquaculture (29). As one of the most studied species of fish (30, 31), information about zebrafish capacities and interests has the potential to set expectations for fishes in general and can influence fish welfare standards. Moreover, as a member of the *Cyprinidae* family, zebrafish can provide insights into patterns of behavior and motivation for *Cyprinidae*, one of the most farmed families of fishes (32).

By characterizing the exploratory behavior of groups of zebrafish toward novel objects, we hypothesized that initial interest could range from neophobic (manifesting as avoidance and fear responses), to indifference (little to no behavioral changes in response to the objects), to curiosity (exploration of and attraction to the objects). We further distinguished between indiscriminate neophilia and targeted information-seeking. We operationalized indiscriminate neophilia as fleeting and uniform or haphazard interest across objects, i.e., short, irregular, nondifferentiated interest durations. In contrast, we operationalized targeted information-seeking behavior as sustained, differential attention, i.e., the presence of distinctive and consistently high interest for protracted durations of time for only some object presentations.

Pending evidence of targeted information-seeking behavior, we also sought to determine whether there was consistency across tanks as to which objects attracted the most sustained attention. General consistency in object-level attraction across tanks would indicate that some perceptual property of the objects themselves were driving the differential interest. Accordingly, we aimed to select objects that varied in complexity, ecological plausibility, and predator resemblance, allowing us to assess (i) which of these dimensions might produce the most sustained information seeing, (ii) how the introduction of objects may affect welfare, and (iii) what object properties might provide appropriate cognitive enrichment for this species (31).

To achieve these aims, we observed the behavior of groups of zebrafish toward a range of 30 different novel objects presented one at a time in their home tanks. The zebrafish were housed in relatively large (110 L), semi-naturalistic aquaria that could accommodate multiple behaviors, thus providing the fish with a low-stress, free-choice (explore or not) set up that allowed them to engage with a particular novel object or not. In addition to object-directed behavior, we also recorded aggression, shoal cohesion, swimming coordination, and diving behavior during a baseline period without a novel object and while the object was in the tank. Zebrafish social dynamics are sensitive to environmental manipulations (33), with recent empirical work suggesting that free-choice exploration can increase prosocial behavior and decrease diving (34), whereas potential stressors have the reverse effects (35). Together, this suite of behaviors can provide indications of information-seeking motivation in zebrafish as well as preliminary indications as to the valence (positivity/negativity) of the zebrafishes' response to the novel object exploration opportunities.

## Materials and methods

### Ethics statement

All husbandry and experimental procedures were approved by the University of British Columbia's Animal Care Committee, protocol number A14-0119.

### Animals and housing

**Figure 1:**
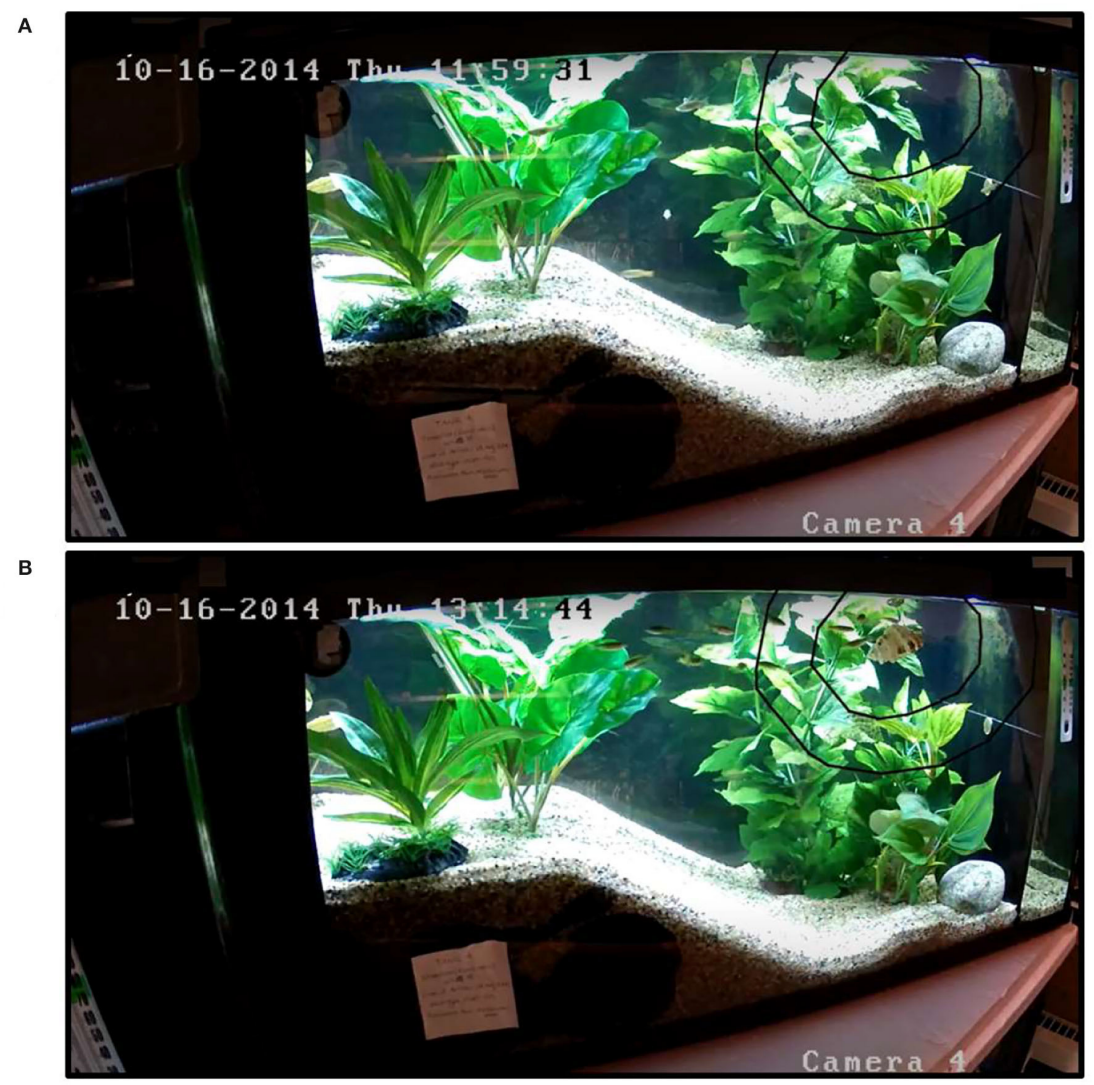
Example zebrafish tank provided from stills of video data collected during **(A)** baseline and **(B)** corresponding object presentation. Two tanks were set-up with this orientation and four tanks were set-up in the reverse direction (slope on the right and deep-end/object presentation area on the left). The baseline period **(A)** was defined as at least 1 h before a novel object was introduced into the tank. The concentric outlines around the object in panel **(B)** are overlain on both videos to indicate the areas within approximately two zebrafish body-lengths from the object and four zebrafish body-lengths from the object. Fish presence in the inner area (two body-lengths) was used for the analyses. Each object-tank combination had its own outline to control for differences in object size and shape so that interest scores could be calculated as the number of fish in the inner area beyond the number observed during baseline: interest score = fish in inner area_object presentation_ – fish in inner area_baseline_.

Sixty adult (>4 months of age) wild-type, short-fin zebrafish (*Danio rerio*) were purchased from a local pet store (Vancouver, BC). Three months prior to the start of the experiment and throughout the experiment itself, fish were housed *via* random assignment into mixed-sex groups of 10 fish per group in stand-alone tanks furnished with sloping gravel substrate, rocks, artificial plants, and wrapped black plastic on three sides to minimize visual disturbance (fish/tank = 10; tank: *n* = 6, 110 L, 30 cm x 91 cm X 40 cm; see Figure 1). Tanks were the experimental unit in this study and were placed on a rack with two tanks per shelf, such that the top two and bottom two tanks had the slope on the right, the middle two tanks had the slope on the left. Zebrafish were fed to satiation with standard flake food (Nutrafin Max Tropical Fish Flakes, Hagen, Canada) twice per day. Room lights were on a 12L:12D schedule with tank lights turning on an hour after room lights and turning off an hour before room lights to graduate light-intensity changes. Water was kept at 26–28 degrees Celsius. Twenty percent water changes were performed once a week, followed by water quality checks 2 days later to confirm minimal ammonia (<0.1 ppm), nitrate (<20 ppm), nitrite (<0.25 ppm), and a pH of 7. After the conclusion of this research, the fish were adopted into private homes.

### Experimental procedures

All procedures were conducted in the home tanks. Over the period of a month on 16 test days, thirty different novel objects (see Appendix) were inserted into the deep end of the tank (see Figure 1), one-at-a-time for a period of 10 min each with at least 30 min between presentations. Objects were fixed to the lid of the tank *via* a clear plastic rod so that they were suspended ~2 cm below the water surface. After positioning the object, the experimenter retreated to a corner of the room out of sight of the fish. Fish behavior was video recorded *via* video cameras (Swann NVR8-7200; resolution: 1000 TVL/1080p) fastened 1 meter in front of each tank.

Video recordings for each tank-object combination were edited to create 100-s clips for each of 3 observation periods: baseline (an hour or more before object presentation), first-100 s (from when the object was placed in the tank for the next 100 s), and final-100 s (the last 100 s before the object was removed from the tank). All videos were overlaid with two outlines indicating (i) the area within two-body lengths of the object and (ii) the area within four-body lengths of the object (see Figure 1). Only the first area (within two body-lengths) was used in the analyses. Thirty objects, six tanks, and three observation periods resulted in 540 video clips; however, 18 clips could not be used due to technical issues with the video, leaving 522 clips for analysis.

Objects (all made of inert and insoluble materials) were selected by human coders in an attempt to capture a range of characteristics along non-orthogonal dimensions of complexity, ecological plausibility, and predator resemblance. Power analysis indicated that a sample of 30 novel objects would allow us to detect correlations of at least 0.50 between object dimensions and zebrafish interest with ~80% power. Eight coders with varying degrees of zebrafish expertise (from none to 10+ years of experience) showed a high degree of inter-rater reliability in their characterization of the objects along these dimensions (Cronbach alpha > 0.85), which allowed us to average their ratings together to create one composite score for each object along each human-generated dimension.

All tanks were exposed to all objects in a pseudo-randomized order across the entire study. The order could not be fully randomized as object presentation periods occurred simultaneously for the six tanks and we only had one version of each object. However, the pseudo-randomization schedule ensured that all objects were presented equally during the beginning, middle, and end of the study.

### Behavioral coding and scores

All behavior was coded from the video clips in a randomized order by observers who were blind to condition and study predictions and trained to a minimum of 0.70 inter-rater reliability. Approach latency was scored as the time in seconds until the first fish entered the inner object area, i.e., approached the object within two body-lengths. Scan samples of the number of fish in the inner area, all social behaviors (agonism, cohesion, and coordination), and diving behavior were recorded every 10 s (ten scores per video clip) and then averaged to create a single score for each tank-object observation period. Agonistic behavior—chasing, charging, fleeing, biting, and lateral displays—was coded as present/absent. Cohesion—the degree to which fish occupied the same space—was coded on a scale from 0 (uniformly spread out) to 4 (tightly clumped). Coordination—the degree to which fish were swimming in the same direction—was coded on a scale from 0 (swimming in all different directions) to 4 (all fish swimming in the same direction). Diving behavior was scored as the proportion of visible fish near the gravel (in the bottom third of the tank) vs. higher in the water column. Previous studies used this methodology and describe it in more detail with video examples [see (34)].

We coded zebrafish behaviors during the first 100 seconds (first-100 s) and the final 100 s (final-100 s) of the 10-min object presentation. We also coded 100 s of behavior during a baseline period, ~1 h before when there was no object present. It is also important to note that these behaviors are not mutually exclusive, do not constitute an exhaustive ethogram, and can, in principle, vary independently of each other—e.g., agonism can increase or decrease along with increased fish presence in the inner area, coordination can increase or decrease along with an increased proportion of fish diving to the lower parts of the water column.

All behaviors scores were calculated for statistical modeling as a difference between the average level of behavior across scan samples for a tank during an object presentation period (either during the first-100 s or the final-100 s) and the average level of behavior across scan samples for that tank during that object's baseline period. For example, if we consider tank 4, object presentation number 4 and hypothetically pose that we found (i) an average of 2.2 fish in the inner area during baseline (across the 10 scan samples), (ii) an average of 7.7 fish in the inner area during the first-100 s (across the 10 scan samples), and (iii) an average of 3.3 fish in the inner area during the final-100 s (across the 10 scan samples). These data would yield an interest score of 5.5 for the first 100-s (interest score = 7.7–2.2) and 1.1 for the final-100 s (interest score = 3.3–2.2). In general, interest scores could range continuously from −10 to +10 because the average number of fish in the inner area could range from 0 to 10. Agonistic behavior scores and diving behavior scores could range continuously from −1 to +1 because they were calculated as proportions (i.e., 0 to 1; proportion of scan samples with agonistic behavior and proportion of fish in the bottom of the water column). Cohesion scores and coordination scores could range continuously from −4 to +4 because they were coded on scales from 0 to 4.

### Data analyses

We treated tank as the unit of analysis (*n* = 6). With repeated measures of tanks and objects, our general approach to account for pseudoreplication was to apply multilevel models (identity link, Gaussian error structure) to control for crossed-random effects of both tank and object (36, 37). Each model contained the continuous outcome variable of interest (e.g., interest score, agonistic behavior score), at least one fixed effect to test study predictions (e.g., object presentation period), and had crossed-random effects of tank ID and object ID. This modeling approach allowed us to treat the tank as the unit of analysis while also controlling for repeated sampling of tanks and objects across the study duration. We used the Satterthwaite method to calculate approximate degrees of freedom for the t-statistics of the fixed-effects null-hypothesis testing.

To assess changes to behavior during object presentation periods vs. the baseline period, outcome variables included: interest scores, social behaviors, and diving behavior. To determine the consistency of differential sustained interest, we modeled interest scores during the final-100s as the outcome and the corresponding interest scores during the first-100 s as a fixed effect predictor (with tank and object as crossed-random effects). Variability in interest was assessed with Likelihood Ratio Tests (36, 37) comparing a base model with tank but not object (or object but not tank) as random effects to an augmented model that included both object and tank as crossed-random effects. These model comparisons test whether object (or tank) ID can account for a significant proportion of the variability in interest-scores.

All data manipulation, plotting, and statistical analyses were conducted with R (38) in RStudio (39), using the following packages: tidyverse (40), psych (41), gridExtra (42), lme4 (43), and lmerTest (44).

## Results

Zebrafish began exploring objects almost immediately with a median approach latency of <1 s, a maximum approach latency of 48 s, and 95% of all first approaches occurring within 6 s.

**Figure 2:**
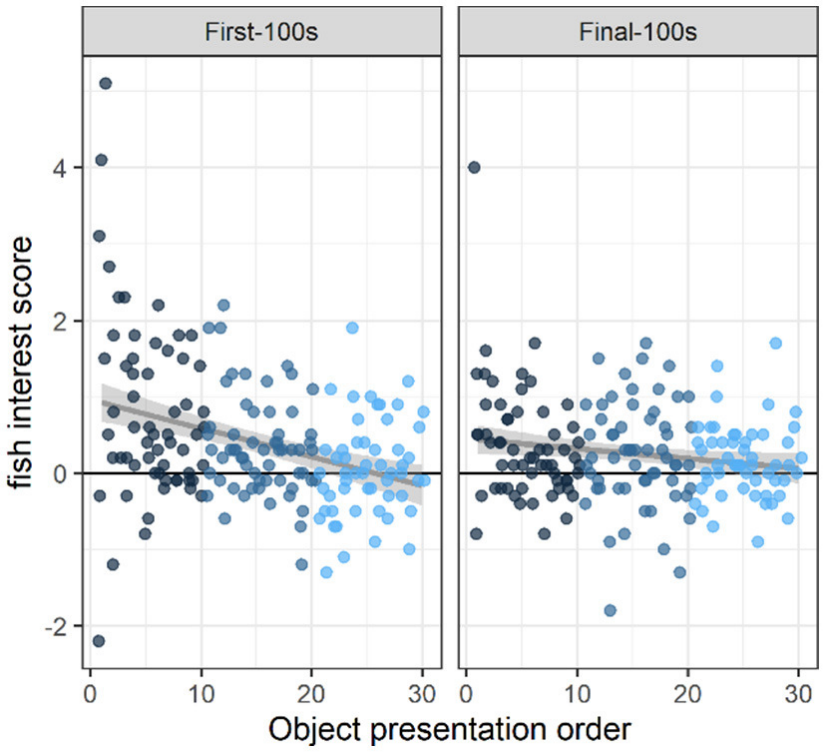
Interest across novel object presentations. Fish interest scores were calculated as the difference in the average number of fish in the inner area (within two body-lengths of the object) during the object presentation period vs. a baseline period when there was no object present. These data show that fish were significantly attracted to the novel object at the beginning of the study (i.e., the first 10 objects they saw) during the first-100 s of an object presentation (*p* < 0.0001) *and* during the final-100 s of object presentation (*p* < 0.01). By the end of the study, (i.e., the last 10 objects they saw), there was no strong evidence of overall attraction to (or avoidance of) the novel object area (*p's* > 0.05). Across presentations, therefore, interest in the novel objects decreased during both periods (first-100s: *p* < 0.0001; and final-100s: *p* < 0.02). Dots indicate the number of fish in the object area above the average number of fish in the object area during baseline. Dots are colored according to object presentation order (dark blue: 1–10; medium blue: 11–20; light blue: 21–30) and are jittered slightly (random noise added to avoid overlapping). Gray lines and shaded areas represent the best linear fit and its 95% Confidence Interval.

At the beginning of the study (i.e., object presentations 1–10), fish were in the object area (within two body-lengths of the object) more often than expected above baseline, during both the first 100 s and the final 100 s of the 10-min novel object presentation (first-100 s: 0.77 above baseline, 95% Confidence Interval [0.51, 1.03] (henceforth indicated by square brackets only), t(154.66) = 5.77, *p* < 0.0001; final-100 s: 0.36 [0.10, 0.62] above baseline, t(154.66) = 2.67, *p* < 0.01; Figure 2). By the end of the study (i.e., object presentations 21–30), overall fish presence in the object area was indistinguishable from baseline (first-100s: 0.06 [−0.10, 0.22] above baseline, t(128.97) = 0.69, *p* < 0.4; final-100s: 0.16 [0.00, 0.32] above baseline, t(128.97) = 1.93, *p* < 0.06; Figure 2).

**Figure 3:**
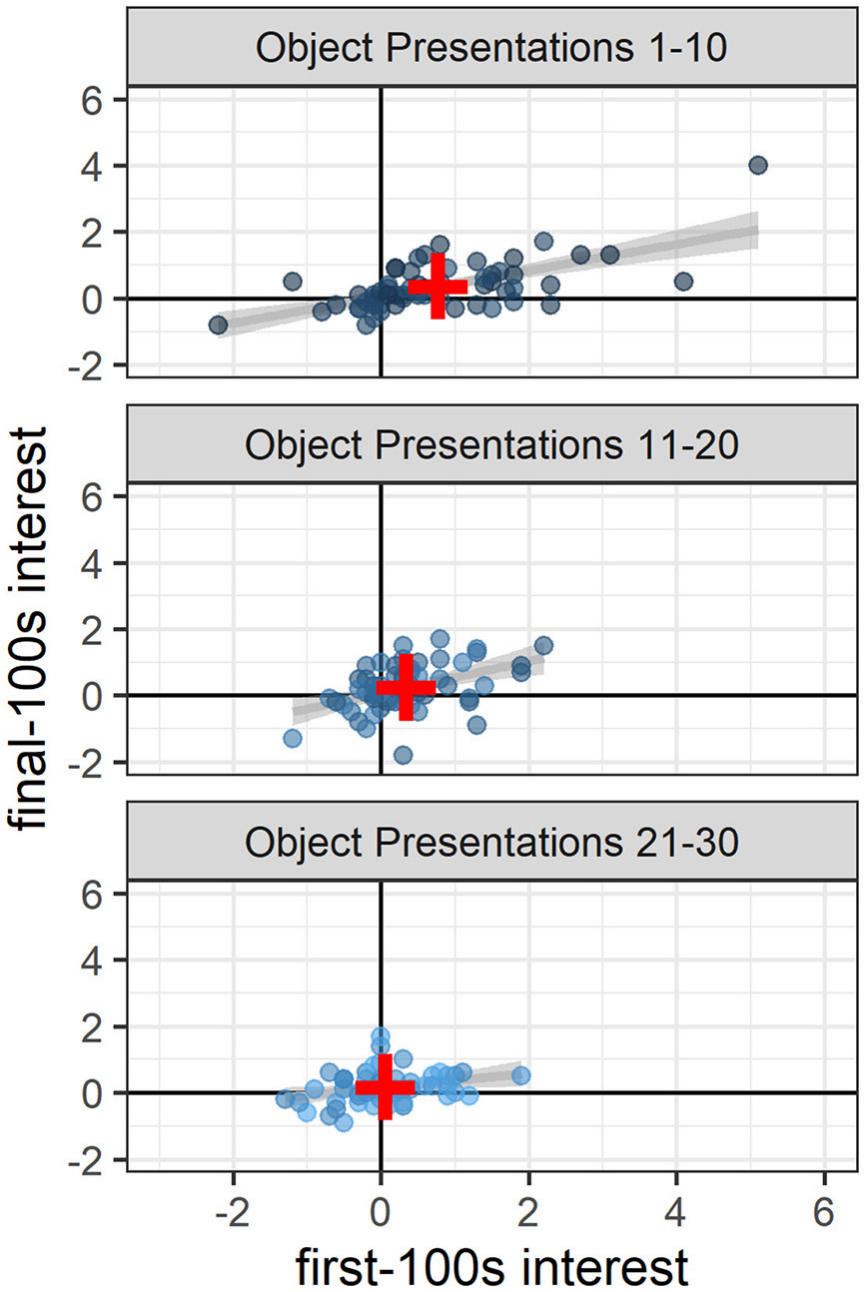
Sustained interest across the 10 min of novel object presentations. Throughout the study, high interest in the first 100 s generally corresponded to high interest in the final 100 s of the 10-min novel object presentations (presentations 1–10: *p* < 0.0001; presentations 11–20: *p* < 0.001; presentations 21–30 *p* < 0.02). Dots indicate the number of fish in the object area above the average number of fish in the object area during baseline and are colored according to object presentation order (dark blue: 1–10; medium blue: 11–20; light blue: 21–30). Gray lines and shaded areas represent the best linear fit and its 95% Confidence Interval. Red plus symbols indicate the center of each bivariate distribution (see online version for color).

High interest during the first 100 s of a novel object presentation generally corresponded to higher interest during the final 100 s of that presentation, a pattern that remained significant throughout the study (object presentations 1–10: 0.40 [0.28, 0.52], t(58.00) = 6.36, *p* < 0.0001; object presentations 11–20: 0.51 [0.27, 0.75], t(55.62) = 4.19, *p* < 0.001; object presentations 21–30: 0.26 [0.06, 0.46], t(49.31) = 2.62, *p* < 0.02; Figure 3).

**Figure 4:**
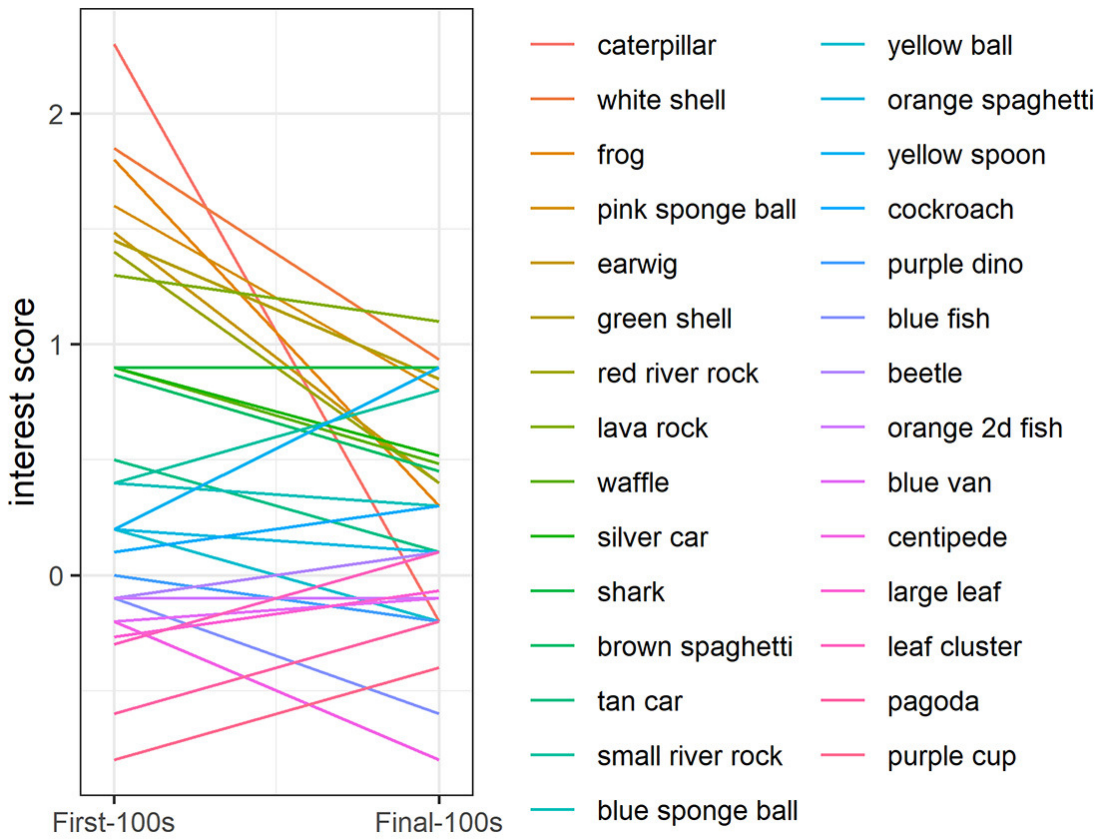
Variability in object interest at the beginning of the study. During the first 10 object presentations, object ID explained a significant proportion of the variance in zebrafish behavior (*p* < 0.01). Interest was quantified as fish presence in the object area (within two body lengths of the novel object) above a baseline period when there was no object present. Colored lines represent object interest scores at the beginning of the study averaged across tanks. The line colors and order of the novel objects in the legend correspond to the plot such that the object that appears first in the legend (“caterpillar”, dark-red line), corresponds to the object with the highest interest score in the First-100 s, followed by objects that attracted less interest in the First-100 s and colored lines progressing through the rainbow, ending with the last object in the legend (“purple cup”, magenta line), which corresponds to the object with the lowest interest score in the First-100 s.

In addition to the greater overall interest at the beginning of the study compared to the end of the study, there was also greater *variability* in interest at the beginning compared to the end: The range in interest for presentations 1–10 was −2.2 to 5.1 vs. the range in interest for presentations 21–30 was −1.3 to 1.9 (Figure 3). At the beginning of the study (object presentations 1–10), object ID accounted for 11% of the variability in interest, with some objects consistently attracting more attention and some objects consistently attracting less attention than other objects [Likelihood Ratio Test: χ2(1) = 7.08, *p* < 0.01; Figure 4]. Tank ID accounted for 21% of the variability in interest [Likelihood Ratio Test: χ2(1) = 15.30, *p* < 0.0001], leaving 68% of the variability in object interest unexplained. None of the human-scored dimensions of object characteristics—complexity, ecological plausibility, predator-resemblance—explained variability in zebrafish interest during this period (*p*'s > 0.4). At the end of the study (object presentations 21–30), neither object ID nor tank ID accounted for the (relatively small) variability in interest (*p*'s > 0.2).

The experimental procedures of introducing of novel objects altered zebrafish social behavior and diving behavior, but unlike interest, these effects remained consistent throughout the study. Compared to baseline: agonistic behavior decreased during the first 100 s (-0.26 [-0.32,−0.20], t(13.73) = 7.86, *p* < 0.0001) and returned to baseline levels by the final 100 s (−0.01 [−0.07, 0.05], t(13.73) = 0.21, *p* > 0.8); cohesion increased during the first 100 s (0.47 [0.33, 0.61], t(5.91) = 7.04, *p* < 0.001) and returned to baseline by the final 100 s (0.05 [−0.09, 0.19], t(5.91) = 0.80, *p* > 0.4); and coordination also increased during the first 100 s (0.68 [0.54, 0.82], t(6.31) = 10.31, *p* < 0.0001) and returned to baseline by the final 100 s (0.13 [−0.01, 0.27], t(6.31) = 2.05, *p* < 0.09).

Throughout the study, diving behavior decreased during the first 100 s of object presentations (−0.26 [−0.30, −0.22], t(15.22) = 10.46, *p* < 0.0001) and returned to baseline by the final 100 s (-0.03 [−0.07, 0.01], t(11.90) = 1.37, *p* > 0.1).

Importantly, at the beginning of the study (object presentations 1–10), changes in social behavior and diving behavior corresponded to zebrafish interest: novel object presentations that elicited greater interest (vs. less interest) corresponded to decreased aggression (−0.06 [−0.10, −0.02], t(107.40) = 2.42, *p* < 0.02), increased cohesion (0.10, [0.02, 0.18], t(92.22) = 2.64, *p* < 0.01), increased coordination (0.08 [0.00, 0.16], t(67.24) = 2.02, *p* < 0.05), and decreased diving (−0.07 [−0.13, −0.01], t(30.99), = 2.10, *p* < 0.05). At the end of the study (object presentations 21–30), the linkage between interest scores and these other behaviors was no longer significant for any behavior (all *p*'s > 0.1).

## Discussion

Across the study, zebrafish readily approached novel objects introduced into their home-tanks with a median approach latency of 1 s. For comparison, a zoo study investigating curiosity across several taxa (sheep, tortoises, penguins, kangaroos, cockatoos, and lemurs) reported that latency to orient to novel objects ranged from 25 s to over 15 min, with many taxa never coming within two body-lengths of some objects (10).

At the beginning of the present study, *i.e*., object presentations 1–10, we found evidence for sustained information-seeking: differential, prolonged (up to 10 min), interest consistently displayed toward some objects and not toward others. During this period, we found that interest varied systematically within tank such that objects that attracted high attention during the first 100 s, typically continued to attract above-average attention nearly 10 min later. Further, object ID accounted for 11% of the variability in interest across tanks, indicating that there was some object property driving the zebrafish interest behavior. This type of differential, object-driven sustained attention is more consistent with information seeking than it is with sensation-seeking or indiscriminate neophilia.

None of the dimensions coded by human observers— complexity, ecological plausibility, predator-resemblance—predicted differential zebrafish behavioral responses to the objects. One possible interpretation of this null result is that while we classified objects based on their visual characteristics from a human perspective, the zebrafishes' interest may have been determined by multiple sensory features as perceived by them, including, for example, chemosensory properties and fluid dynamics [zebrafish are sensitive to water flow; (45)]. Thus, it is possible that the theories about what predicts greater curiosity may have been borne out from the fishes' perspective: the objects with the most complex and most unusual features in multi-sensory space in zebrafish perception could have attracted the most attention from the zebrafish. For instance, across tanks, a small white shell and a large neon pink artificial ball both attracted a great deal of attention at the beginning of the study, despite being markedly different in human-coded complexity, ecological-plausibility, and predator-resemblance, and different in terms of shape, size, color, and material. In a multisensory space from a zebrafish's perspective, however, perhaps the white shell and pink sponge ball were equivalently unusual or complex.

Determining what parameters drive intrinsically motivated exploratory behavior in zebrafish is an area for future research, but as indicated in the present results, serial presentations of multiple static objects (the design employed for this study) is unlikely to yield the best data. At the end of the study, approaches remained fast, but interest was relatively uniform (variability in interest between object presentations was low), fleeting (interest did not extend throughout even the first 100 s of object presentation), and any variability that did exist was not driven by object characteristics. In other words, by the end of a serial presentation of 30 novel static objects (by object presentations 21–30), there was little evidence for information-seeking, instead the pattern of behavior was more consistent with sensation-seeking.

Importantly, objects were presented in a pseudo-randomized order, ensuring that across tanks, the objects during the early period were the same as the objects at the end of the study. As such, the drop-off in information seeking cannot be due to some object-level feature. Instead, the loss of interest could be due to some form of habituation to the task. At the beginning of the study, the task itself contained new information as the zebrafish learned what to expect from the procedures. By the end of the study, there was less information to be gained about the situation itself, with the only unknown aspects being those associated with the stationary, nonmanipulable, static objects themselves. It is possible, therefore, that at the beginning of the study, one source of the zebrafishes' curiosity was figuring out what to expect with the task, including what the object itself might do or not do and what could or could not be done with the object. If so, introducing more dimensions of variability into the study procedures—e.g., using objects or images that move, change, or are interactive—might facilitate continued information-seeking behavior throughout repeated presentations.

In sum, these results show that in addition to neophilia, zebrafish engage in behaviors that are consistent with the motivation for information gain. That such a motivation exists in zebrafish falls into a long tradition of animal behavior research pointing toward the deep evolutionary roots of an intrinsic motivation to explore, learn, and make sense of the world and suggests the possibility that zebrafish, like other species, may find information acquisition rewarding (12, 21–23, 46).

### Welfare and cognitive enrichment

Neophilia in the absence of sustained attention, as observed at the end of the study, could be interpreted as a sign of boredom with the task and/or boredom in general. Despite being housed in semi-natural tanks, daily life in these tanks was likely less varied and less cognitively stimulating than it would be for a zebrafish living in the wild (33, 47). As such, it is possible that the zebrafish in this study were under-stimulated, i.e., experiencing a restriction of behavioral opportunities. Future research could look to establish the evidence of boredom in zebrafish by, for example, assaying for distorted time perception and tolerance of or even attraction to mildly aversive experiences, both of which are considered evidence of boredom in other species (27, 28).

Nonetheless, evidence that zebrafish have the capacity to engage with information-seeking for its own sake suggests that certain forms of cognitive stimulation could be beneficial zebrafish enrichment. Providing free-choice cognitive stimulation opportunities is known to increase welfare in other species (48) and may contribute to positive welfare (49). Crucially, the present work shows that not all novel objects were equally captivating to zebrafish, so not all novel objects are likely to be equally suitable candidates for enrichment. Moreover, the objects themselves, even those that appeared to stimulate information-seeking for the tanks that saw them at the beginning of the study, were not able to produce an information-seeking response for the tanks that saw them at the end of the study. As such, while these results point toward the possibility that zebrafish could benefit from long-term cognitive enrichment, the form that enrichment should take was not identified in the present work. Dynamic, changeable, and interactive objects or images (e.g., providing videos or vistas) may be more promising targets.

Along these lines, it is worth noting that the zebrafish in the present study were provided with environmental complexity that is more typical of home aquaria than laboratories, where small, barren tanks are currently the norm (30). Given that barren housing is aversive to zebrafish (45, 50) and detrimental to their welfare and cognition (31, 51), some of the work identifying sensation-seeking behavior in zebrafish, including impulsivity (52) and drug-seeking behavior (53), may be a product of these barren abnormal environmental conditions rather than a feature of normal zebrafish behavior. Similarly, if the present research had been conducted with zebrafish housed in the barren tanks typical of most laboratory research (30), we might not have been able to detect intrinsic information-seeking behavior, which is understood to depend on non-aversive housing conditions and low-stress conditions (21, 23, 54). As such, the implication of this work for barren-housed zebrafish is the “in principle” observation that some form of cognitive enrichment may be worth considering given zebrafish capacities and interests; the form that the cognitive enrichment takes is likely to be conditional on background housing conditions.

### Positive welfare and emotional valence

The added value of a positive welfare approach is that it brings a “full life” perspective to animal welfare research and animal protection efforts (55). In the past, much of animal welfare science focused on alleviating suffering, rather than on considering and promoting positive states. Recent work across the animal kingdom, including promising indications in fishes as well (56), has established that while the alleviation of suffering is an urgent priority requiring immediate attention, on its own, a sole focus on suffering alleviation does not produce long-term welfare. For instance, as mentioned above, in the absence of opportunities for positive engagement, animals can slide into inescapable boredom and poor welfare (27, 28). Thus, in addition to an absence of negative experiences, ensuring even neutral animal welfare requires some positive experiences, including perhaps, opportunities for cognitive engagement and exploration (49).

Information-seeking behavior is consistent with a positive welfare framework, but it is unclear from the exploratory behavior alone whether the zebrafish in this study experienced positive affect as a result of the novel object exploration opportunities. The consideration of affective behavioral responses (i.e., positively/negatively valenced emotion) in zebrafish is now recognized as an important area of inquiry (57) and can be informed by examining their social and diving behavioral responses.

In the current study, the suite of social and diving behavioral changes observed in response to the presentation of novel objects is consistent with positive affect, or at a minimum, the absence of negative affect. Compared to a baseline period an hour before the introduction of an object, the first 100 s of object exposure caused agonistic and diving behavior to drop and shoal cohesion and group coordination to increase. In zebrafish, diving behavior is recognized as an important indicator of stress and negative affect (58) and has been observed in these fish in response to potentially stressful husbandry procedures (35). Despite the possibility that the fish could have responded similarly to the present procedures (i.e., responded by diving to the bottom of the water column), we found no evidence of increased diving. Indeed, we observed *less* diving in the presence of novel objects compared to a baseline period 1 h earlier. The suppression of diving behavior compared to baseline shows, at a minimum, that the free-choice exploration opportunity of novel objects did not produce an overriding stress response in the zebrafish. Decreased aggression and increased affiliative behaviors, especially group synchrony, are often taken as indicators of positive emotions in other species (59, 60), with similar patterns found in zebrafish as well (61). Accordingly, while some of these behaviors are not good indicators of valence on their own (e.g., increased cohesion can also be a stress response in zebrafish), as a group, the overall pattern of behavioral changes during the first 100 s of object presentations is not consistent with a stress response and could indicate the presence of positive affect.

Importantly, at the beginning of the study, when there was the strongest evidence for information-seeking, greater exploration of the objects corresponded to greater signs of positive affect (or low negative affect) in these additional behavioral measures. In other words, when fish were potentially engaged in high levels of object-driven information-seeking, they showed the least diving behavior and the most prosocial behavior. Later in the study, when there was less evidence for object-driven information seeking and only evidence for neophilia or perhaps even arousal or stimulation from the experimental procedures themselves, the correspondence between exploration and the other behaviors was no longer evident.

Finally, alterations in diving and social behaviors only occurred during the first 100 s of object presentation and returned to baseline by the final 100 s of the 10-min object presentation period. As a whole, this pattern of results suggests that information-seeking opportunities may have temporarily increased positive affect in zebrafish, but that this effect was short-lived and may not have improved welfare overall. Future work could investigate how long-term cognitive stimulation opportunities may affect overall welfare outside the immediate presentation periods, potentially with a between-tank experimental design in which only some tanks receive cognitive engagement opportunities while others are maintained in baseline conditions or are exposed to the same object repeatedly.

## Conclusion

We found evidence that, in addition to being neophilic, zebrafish engage in sustained information-seeking behavior. As such, this study provides justification for future work exploring the extent to which fish might find cognitive stimulation rewarding and the role cognitive engagement cognitive engagement plays in their overall welfare. Further investigation into cognitive enrichment for fish will contribute to the growing literature on positive wellbeing, which has become an important topic of research across disciplines. Within the human literature, positive psychology theories have proved to be generative (62) and in the animal literature, a similar pattern is beginning to emerge (63–66). Extending the existing research exploring whether and how fish experience pain (67–69), the present research contributes evidence that fish also have the capacity for positively valenced experiences and are thus good candidates for future research on positive welfare.

Fish welfare is threatened by multiple forms of human activity—from industrial fishing to farming to scientific research to entertainment. There is an urgent need to understand and protect their wellbeing, yet comparatively little species-specific research investigates fish welfare (32) and fish are generally perceived as less deserving of enrichment than other vertebrates (70). One source of this discrepancy may be the general perception that fish are “primitive” or “lower” vertebrates, a notion that is demonstrably false—as a group, fish are just as evolved as any other extant animal taxa and are cognitively sophisticated (68, 71), with some species of fish even outperforming primates on cognitive tests (72) and passing the mirror-self-recognition test (73, 74). The misclassification of fish as somehow inferior to other vertebrates suggests that research on positive welfare in fishes may also have a special role to play in changing the narrative around what fishes can experience and, therefore, what sort of care and protections they require.

Fishes are also some of the most studied organisms in modern science, but they are rarely studied for their own sake. Much of the research involving fish instead focuses on modeling human biological processes [e.g., gene expression in the brain (75)], testing general theories in behavioral biology [e.g., Optimal Foraging Theory (76)], or determining efficient ways to increase farming and fisheries production. In compliment and in contrast to these anthropocentric forms of fish research, adopting a fish-centric research agenda can facilitate unique basic science contributions (e.g., evolutionary patterns of curiosity across the animal kingdom), ethical insights (fish are capable of positive experiences and may suffer from boredom and other forms of poor welfare in their absence), and practical solutions [e.g., cognitive stimulation may be a valuable source of enrichment for fish; (56)]. Moreover, studying fish in natural or semi-natural conditions can help elucidate the degree to which barren laboratory housing induces abnormal biological states that may reduce the generalizability of data collected.

In conclusion, while we did not find that zebrafish follow the same patterns of curiosity as those found in previously studied (terrestrial) animals, we did find evidence that they find some objects to be more interesting than others. As these exploration opportunities decreased agonistic and diving behavior while also increasing affiliative behavior, it is possible that zebrafish, like many other species, benefit from activities that engage their cognitive abilities and preferences. This research builds on our understanding of the determinants and consequences of curiosity across species and opens new avenues of investigation regarding the role that exploration and learning play in the lives and welfare of fishes.

## Data availability statement

The raw data supporting the conclusions of this article will be made available by the authors, without undue reservation.

## Ethics statement

All husbandry and experimental procedures were approved by the University of British Columbia's Animal Care Committee, protocol number A14-0119.

## Author contributions

All authors listed have made a substantial, direct, and intellectual contribution to the work and approved it for publication.

## Appendix

**Table A1:** Novel object characteristics and photos.

**Object and photo**		**Object and photo**		**Object and photo**	
Beetle C: 3.88 PR: 0.5 EP: 0.5		Frog C: 4.62 PR: 1 EP: 0.74		Purple dinosaur C: 4.12 PR: 0.62 EP: 0	
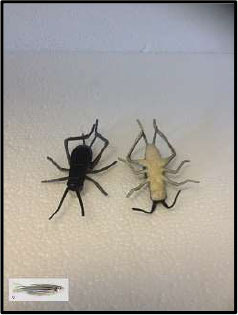
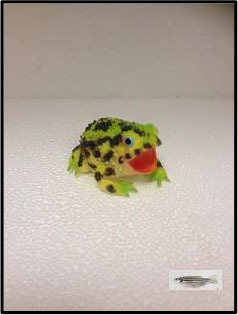
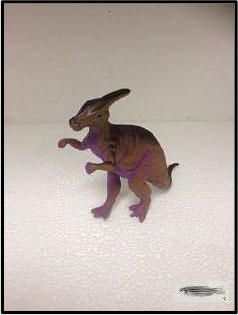
Blue fish C: 2.12 PR: 0.5 EP: 0.5		Green shell C: 3.38 PR: 0 EP: 1		Red river rock C: 1.25 PR: 0 EP: 1	
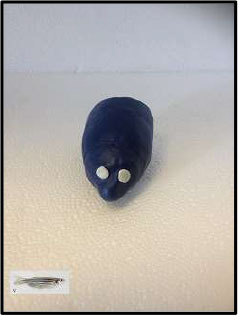
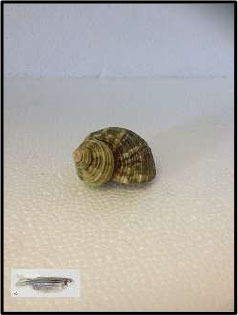
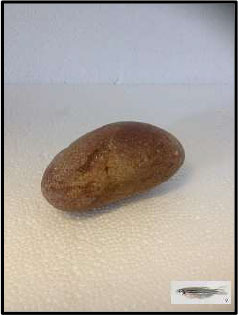
Blue sponge C: 3.75 PR: 0.12 EP: 0		Large leaf C: 2.62 PR: 0 EP: 1		Shark C: 3.88 PR: 1 EP: 0.75	
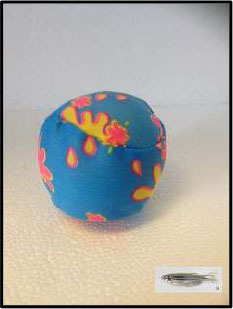
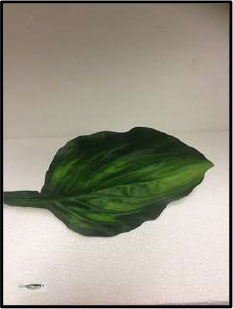
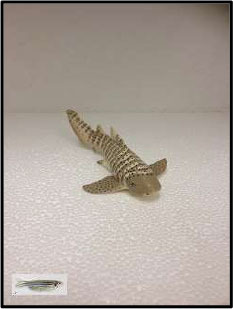
Blue van C: 4 PR: 0.38 EP: 0		Lava rock C: 2.75 PR: 0 EP: 1		Silver car C: 3.25 PR: 0.25 EP: 0	
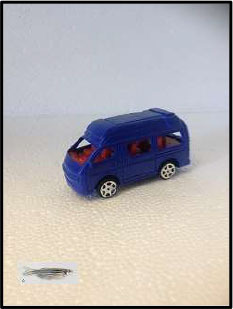
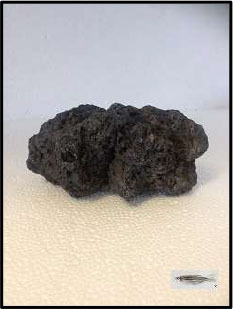
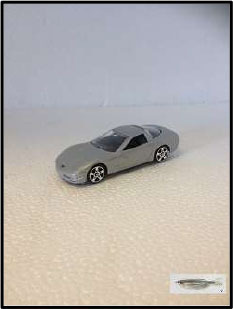
Brown spaghetti C: 3.12 PR: 0 EP: 0.5		Leaf cluster C: 3.38 PR: 0 EP: 0.88		Small river rock C: 1.5 PR: 0 EP: 1	
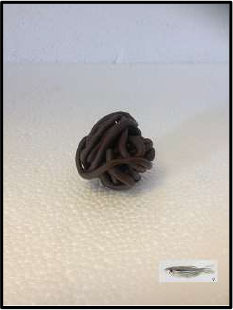
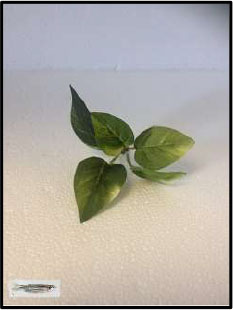
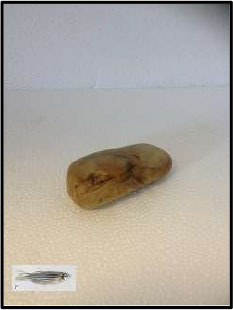
Caterpillar C: 2.62 PR: 0 EP: 0.5		Orange 2d fish C: 3.5 PR: 0.75 EP: 0.25		Tan car C: 4 PR: 0.12 EP: 0	
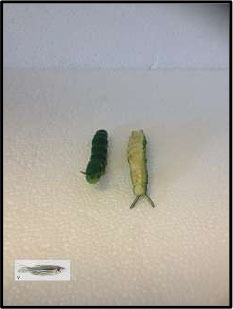
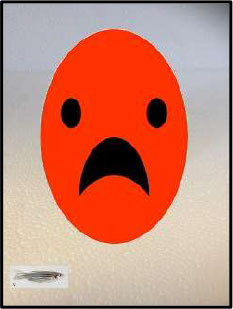
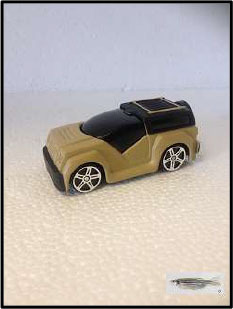
Centipede C: 3.88 PR: 0.12 EP: 0.38		Orange spaghetti C: 3.25 PR: 0 EP: 0.38		Waffle C: 2.88 PR: 0 EP: 0	
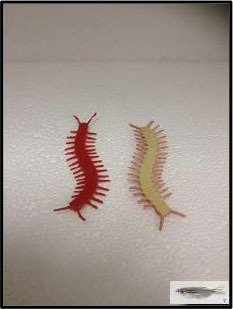
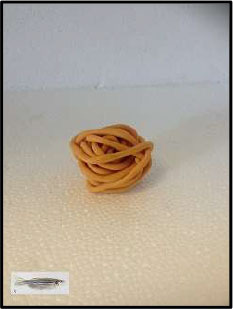
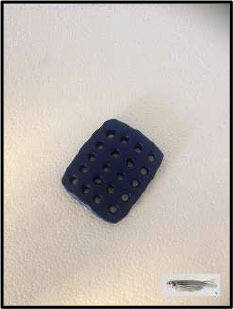
Cockroach C: 4.25 PR: 0.25 EP: 0.75		Pagoda C: 3.5 PR: 0.38 EP: 0.12		White shell C: 3.25 PR: 0.12 EP: 0.75	
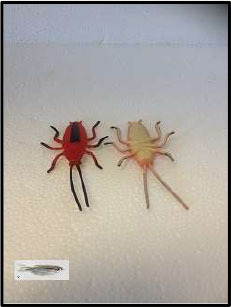
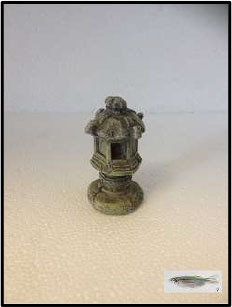
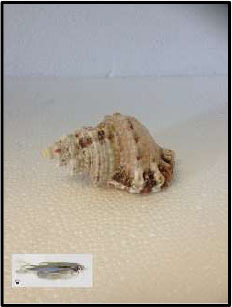
Dark shell C: 3.88 PR: 0.12 EP: 1		Pink sponge ball C: 4 PR: 0.12 EP: 0.12		Yellow ball C: 1.5 PR: 0 EP: 0.12	
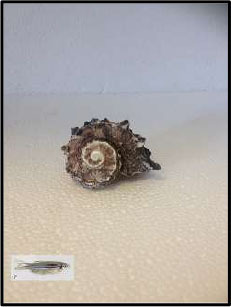
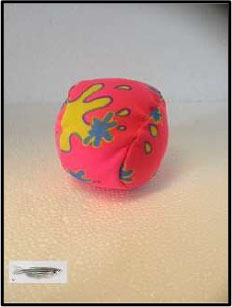
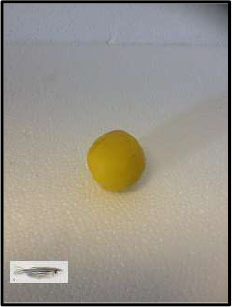
Earwig C: 3.5 PR: 0.12 EP: 0.5		Purple cup C: 2 PR: 0.14 EP: 0.14		Yellow spoon C: 2.38 PR: 0.12 EP: 0	
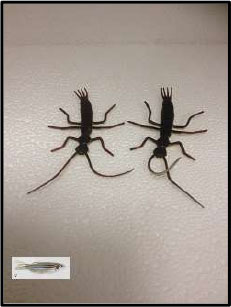
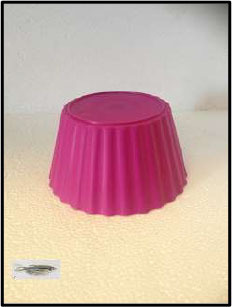
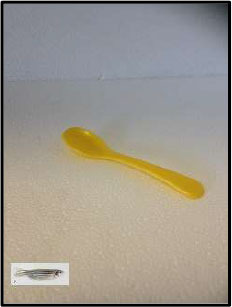

A list of the 30 novel objects used for this study (presented in a pseudo-randomized order across tanks) and the human-coded dimensions of interest: C refers to complexity (visual complexity of the object average score on a scale of 1 to 5), PR refers to predator-resemblance (the degree to which the object resembled a predator of zebrafish average score on a scale of 0 to 1), and EP refers to ecological-plausibility (the degree to which the object represented something a zebrafish might plausibly encounter in its natural environment average score on a scale of 0 to 1). These dimensions were generated by eight human coders that together had a Cronbach alpha of greater than 0.85 agreeability for each of these dimensions. NB: The object identified as “Orange 2d fish” is a rendering of the image that was printed on a sheet of paper and then laminated before being presented to the fish. Small white rectangles within each picture represent an estimate of a zebrafish's size in relation to the object.
